# Antioxidant and Antityrosinase Activity of *Flemingia macrophylla* and *Glycine tomentella* Roots

**DOI:** 10.1155/2012/431081

**Published:** 2012-09-10

**Authors:** Bor-Sen Wang, Lih-Jeng Juang, Jeng-Jer Yang, Li-Ying Chen, Huo-Mu Tai, Ming-Hsing Huang

**Affiliations:** ^1^Department of Food Science & Technology, Chia Nan University of Pharmacy and Science, 60 Erh-Jen Road, Section 1, Jen-Te, Tainan 717, Taiwan; ^2^Department of Applied Cosmetic Science, Ching Kuo Institute of Management and Health, 336 Fu-Hsing Road, Keelung 203, Taiwan; ^3^Department of Pharmacy, Chia Nan University of Pharmacy and Science, 60 Erh-Jen Road, Section 1, Jen-Te, Tainan 717, Taiwan; ^4^Department of Cosmetic Science, Chia Nan University of Pharmacy and Science, 60 Erh-Jen Road, Section 1, Jen-Te, Tainan 717, Taiwan

## Abstract

The antioxidant and antityrosinase activities of the water extract of *Flemingia macrophylla* root (WEFM) were investigated. The results showed that WEFM exhibited radical scavenging and reducing activities, as well as ferrous ion chelating property. In addition, WEFM also protected phospholipids against oxidation, indicating that WEFM could protect biomolecules from oxidative damage. Meanwhile, in the range of 50–100 **μ**g/mL, the tyrosinase inhibitory activity of WEFM increased with an increase in sample concentration and was superior to that of the water extract of *Glycine tomentella* root (WEGT). A high performance liquid chromatography analysis was used to determine the phenolic components, revealing that daidzin, daidzein, genistin, and genistein were present in WEFM and WEGT. Acting as an antioxidant and a tyrosinase inhibitor, these bioactive constituents could contribute to the protective effects of WEFM. Overall, the results showed that WEFM might serve as a natural antioxidant and tyrosinase inhibitor.

## 1. Introduction

The lipid components in food are susceptible to oxidation, which results in some deleterious effects in the course of food storage, and decrease the quality of the food. They are also known to contaminate the food with harmful substances. At the same time, lipid oxidation induced by reactive oxygen species (ROS) may lead to cellular damage and promote the pathological progression of atherosclerosis, carcinogenesis, and diabetes [1]. Therefore, to decrease oxidation, various antioxidants have been investigated to protect the lipids in foods. Moreover, there is currently an interest in research to replace synthetic antioxidants with natural alternatives for safety concerns. Further, as many of the natural phytochemicals present in plants have been found to exhibit antioxidative capacity *in vivo*, they have attracted much attention as natural inhibitors of oxidation in foods [2]. In addition to lipid oxidation, the browning reaction in foods is another unfavourable effect which decreases its appeal and nutritional value during storage, and the pigment melanin in human skin is a major defense mechanism against the ultraviolet light of the sun; the production of abnormal pigmentation, such as melasma, freckles, and other forms of melanin hyperpigmentation can be a serious aesthetic problem. Reports have indicated that tyrosinase had a primary role in the enzymatic browning reaction in foods during processing by catalyzing the oxidation of phenols and regulating the initial step of melanin production [3]. Therefore, many studies have focused on natural additives in order to decrease the browning progression in skin whitening or food production [4]. In addition to food browning, tyrosinase activation and further melanin generation would eliminate the glutathione levels and decrease the antioxidant capacity in foods [5, 6] as well as promote the progression of melanin hyperpigmentation in different skin diseases [7, 8].

Phytochemical-rich natural products, which have a complex array of antioxidants, could exhibit protective effects against oxidative stress in different models. For example, herbal teas are one of the oldest beverages in Southeast Asia, such as China, Taiwan, Vietnam, and Philippines. Recently, a herbal tea prepared from the roots of *Flemingia macrophylla* (FM) or *Glycine tomentella* (GT) is available in teabags on the shelves of supermarkets in Taiwan Island and the south of China. The roots of FM and GT, which were misused or substitutionally used on the market, have long been used to treat rheumatism, arthritis and muscle pains, and so forth [9]. Both contain daidzin, daidzein, fleminone, qurateacatechin, genistin, and genistein as their bioactive constituents [10, 11]. The local herbalists claim that these two functional teas have similar pharmacological activities in analgesic, antipyretic, and antirheumatoid effects. Although the roots of GT and FM have shown some physiological effects [12–14], there are few studies focusing on their effects on tyrosinase and quantitative antioxidant activity. Consequently, the objective of the present study was to determine the effects of aqueous extract of GT and FM roots and their reference compounds against the antioxidant and tyrosinase activities.

## 2. Materials and Methods

### 2.1. Materials

1,1-Diphenyl-2-picrylhydrazyl (DPPH), 2,2′-azino-bis-(3-ethylbenzothiazoline-6-sulphonate) diammonium salts (ABTSs), lecithin, potassium ferricyanide, ferrozine, thiobarbituric acid (TBA), and mushroom tyrosinase were purchased from Sigma (St. Louis, MO, USA). Daidzin, daidzein, genistin, and genistein were purchased from extrasynthese (Genay, France). Acetonitrile was of the LC grade (Fisons, Loughborough, England). The roots of *Flemingia macrophylla* and* Glycine tomentella* were obtained from local Chinese herbal medicine stores in Tainan, Taiwan.

### 2.2. Sample Preparation

All dried samples were cut into small pieces and ground into fine powder. Each sample (50 g) was extracted with boiling water (1000 mL) for 30 min, and the filtrate was evaporated to dryness under vacuum. The final dried extracts of roots of *Flemingia macrophylla* and *Glycine tomentella* were named as WEFM and WEGT, respectively. The yield obtained was 23.8% (11.9 g) and 16.6% (8.3 g), respectively.

### 2.3. High-Performance Liquid Chromatography (HPLC) Analysis

A high-performance liquid chromatography (HPLC) was performed with a Hitachi Liquid Chromatography System (Hitachi Ltd., Tokyo, Japan), consisting of two model L-7100 pumps and a model L-7455 photodiode array detector. Samples (10 mg/mL) were filtered through a 0.45 *μ*m filter. The injection volume was 20 *μ*L, and the flow rate was 0.8 mL/min. The separation temperature was 25°C. The column was a Cosmosil 5C_18_-AR (5 *μ*m, 250 × 4.6 mm ID; Nacalai USA, Inc.). The method involved the use of a binary gradient with mobile phases containing (A) phosphoric acid in water (0.1%, *v/v*) and (B) H_2_O/CH_3_CN: 20/80 (*v/v*). The solvent gradient elution program was as follows: 0–5 min, 100–90% A, 0–10% B; 5–20 min, 90–85% A, 10–15% B; 20–25 min, 85–80% A, 15–20% B; 25–30 min, 80–60% A, 20–40% B; 30–40 min, 60–40% A, 40–60% B; 40–50 min, 40–10% A, 60–90% B; 50–60 min, 10–0% A, 90–100% B; finally, 60–70 min, 0% A, 100% B. Based on the plot of the peak-area ratio (*y*) versus concentrations (*x*, *μ*g/mL), the regression equations of daidzin, daidzein, genistin, and genistein and their correlation coefficients (*r*) were as follows: daidzin, *y* = 0.0234*x* + 0.0012 (*r* = 0.9969); daidzein, *y* = 0.0533*x* − 0.0018 (*r* = 0.9970); genistin, *y* = 0.0356*x* − 0.0002 (*r* = 0.9969); genistein, *y* = 0.0469*x* + 0.0029 (*r* = 0.9972).

### 2.4. Determination of Total Polyphenols

Total polyphenols were determined as gallic acid equivalents [15]. Different concentrations of the samples were added with 2 mL sodium carbonate (20% (*w/v*)) individually to a 10 mL volumetric flask. After 5 min, 0.1 mL Folin-Ciocalteu reagent (50% (*v/v*)) was added, and the volumes were made up to 10 mL with H_2_O. After incubation at 30°C for 1 h, the absorbance at 750 nm was measured and compared to the gallic acid calibration curve.

### 2.5. Determination of Total Flavonoid Contents

Different concentrations of the samples were added with 0.1 mL (2-aminoethyl) diphenyl borate (0.2% in ethanol) individually. After 20 min of incubation, the absorbance at 405 nm was measured and compared to a rutin calibration curve [16].

### 2.6. Determination of DPPH Radical Inhibition

The assay was carried out as previously described [17]. Each sample (0.5 mL) was added to 0.5 mL of 0.4 mM DPPH in methanol. The mixture was shaken vigorously and allowed to stand for 30 min; the absorbance of the resulting solution was measured at 517 nm with a spectrophotometer (Hitachi U-2000, Japan). A lower level of absorbance indicated a stronger radical scavenging activity.

### 2.7. Determination of ABTS Cation Radical Inhibition

This assay determined the capacity of samples to scavenge ABTS^•+^ as previously described [18]. The ABTS^•+^ was generated by reacting 1 mM ABTS with 0.5 mM hydrogen peroxide and 10 units/mL horseradish peroxidase in the dark at 30°C for 2 h. After 1 mL of ABTS^•+^ was added to samples, the absorbance at 734 nm was recorded after 10 min.

### 2.8. Determination of Reducing Activity

The reducing power of sample was determined as previously described [19]. Potassium ferricyanide (2.5 mL, 10 mg/mL) was added to samples in phosphate buffer (2.5 mL, 200 mM, pH 6.6), and the mixture was incubated at 50°C for 20 min. Trichloroacetic acid (2.5 mL, 100 mg/mL) was added to the mixture, which was then centrifuged at 1,000 g for 10 min. The supernatant (2.5 mL) was mixed with distilled water (2.5 mL) and ferric chloride (0.5 mL, 1.0 mg/mL), and then the absorbance was read at 700 nm. Higher absorbance of the reaction mixture indicated greater reducing activity. 

### 2.9. Determination of Chelating Activity

The chelating activity of samples on Fe^2+^ was measured as previously described [20]. Each sample (0.6 mL) was reacted with FeCl_2_ (2 mM, 0.2 mL) and ferrozine (5 mM, 0.2 mL) for 10 min, and the spectrophotometric absorbance was determined at 562 nm. A lower level of absorbance indicated a stronger chelating activity.

### 2.10. Determination of Liposome Oxidation

Lecithin (500 mg) was sonicated in an ultrasonic cleaner (Branson 8210, Branson ultrasonic Corporation, Danbury, CT, USA) in phosphate buffer (50 mL, 10 mM, pH 7.4) for 2 h in an ice-cold water bath. The sonicated solution, FeCl_3_, ascorbic acid, and samples (0.2 mL) were mixed to produce a final concentration of 3.12 *μ*M FeCl_3_ and 125 *μ*M of ascorbic acid. The mixture was incubated for 1 h at 37°C by the thiobarbituric acid (TBA) method [21]. The absorbance of the sample was read at 532 nm against a blank, which contained all reagents except lecithin. A lower level of absorbance indicated stronger protective activity.

### 2.11. Determination of Tyrosinase Activity

The mushroom tyrosinase was used for the bioassay. The inhibition on tyrosinase-catalyzed oxidation of L-DOPA was determined as previously described [22]. The reaction mixture contained samples, phosphate buffer (0.8 mL, 25 mM, pH 6.8), and L-DOPA (final 3.8 mM), then mushroom tyrosinase (0.1 mL, final 250 units/mL) was added into the mixture to measure the initial rate as a linear increase in the absorbance at 475 nm for 5 min. The reaction was performed at 25°C. The value in the absence sample was represented as the control:

(1) $$\begin{array}{c} \text{Inhibition}\;\;\left(\%\right)=\left(1-\frac{\Delta {\text{OD}}_{475}\;\;\text{in}\;\;\text{sample}}{\Delta {\text{OD}}_{475}\;\;\text{in}\;\;\text{control}}\right)\times 100\%. \end{array}$$

### 2.12. Statistical Analysis

Statistical analysis adopted in this study involved the use of the Stat View statistical package (SAS institute Inc.). Analysis of variance was performed by ANOVA procedures. Significant differences between means were determined by Duncan's multiple range tests at a level of *P* < 0.05.

## 3. Results 

**Figure 1:**
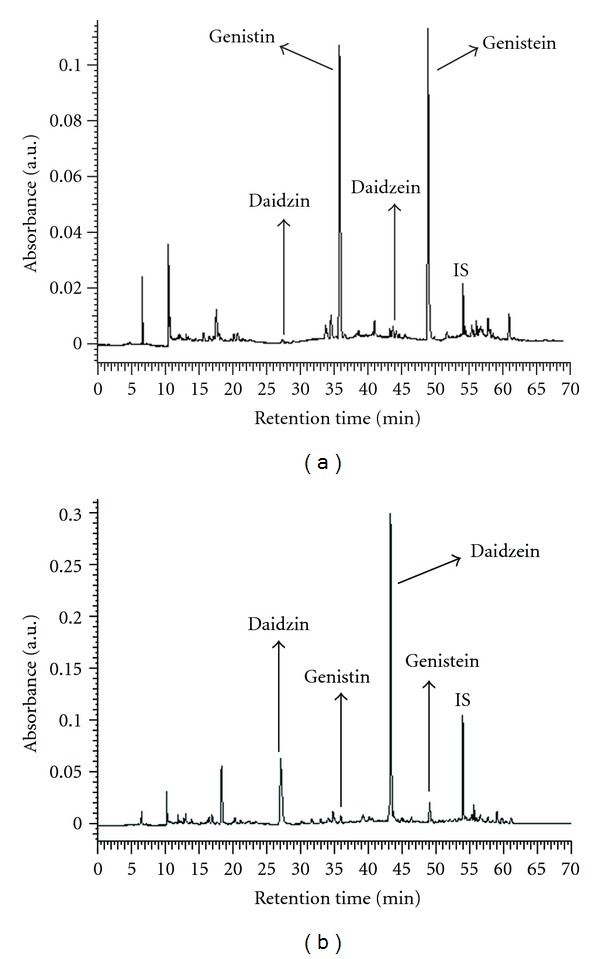
HPLC chromatograms of (a) water extract of *Flemingia macrophylla* root (WEFM) and (b) water extract of *Glycine tomentella* root (WEGT).

Previous studies have indicated that the free radical scavenging activities observed in FM and GT may be due to various natural polyphenols such as daidzin, daidzein, genistin, and genistein which displayed antioxidative effects [23]; thus, daidzin, daidzein, genistin, and genistein were selected as fingerprint markers for HPLC analysis of WEFM and WEGT, and the chromatograms were shown as in Figure 1. Comparing the amounts of four marker phenolic compounds of WEFM and WEGT, the major bioactive constituents in WEFM are genistein and genistin (Figure 1(a)). On the other hand, WEGT contained daidzin and daidzein as its major constituents (Figure 1(b)).  Table 1 shows the concentrations of the four marker compounds in WEFM and WEGT. The relative amounts of the four phenolic compounds were in the order of genistein (8.87 mg/g extract) > genistin (8.20 mg/g extract) > daidzein (0.33 mg/g extract) > daidzin (0.25 mg/g extract) in WEFM and daidzein (4.23 mg/g extract) > daidzin (1.34 mg/g extract) > genistein (0.21 mg/g extract) > genistin (0.16 mg/g extract) in WEGT, respectively. Moreover, the amounts of the genistin and genistein present in WEFM were greater than those found in WEGT. Further, many reports revealed that the physiological function of natural foods could be attributed to their polyphenolic components [24, 25]. Since the total polyphenol and flavonoid contents of natural products were regular indexes of their antioxidant activity, the gallic acid and rutin equivalents were determined in WEFM and WEGT, respectively.  Table 1 shows that the levels of polyphenols were 96.47 and 70.41 mg gallic acid equivalents/g extract, and those of flavonoids were 52.76 and 37.91 mg rutin equivalents/g extract, in WEFM and WEGT, respectively.

Table 2 shows the DPPH radical scavenging activity of WEFM and WEGT. IC_50_ value of WEFM and WEGT was 65.28 *μ*g/mL and 298.66 *μ*g/mL, respectively. The ABTS cation radical inhibition assay, which has been demonstrated to evaluate the total antioxidant capacity of natural products, was also determined. In this study, the water-soluble counterpart of WEFM and WEGT exhibited different scavenging activity against ABTS radical cation in a concentration-dependent manner. Table 2 shows the ABTS radical cation scavenging activity of WEFM and WEGT. IC_50_ value of WEFM and WEGT was 23.05 and 146.23 *μ*g/mL, respectively. These results indicate that the bioactive constituents of the WEFM were potential candidates in scavenging harmful radicals. As shown in Table 2, the antioxidant activities of the four marker compounds were in the order of genistein > genistin > daidzein >  daidzin on ABTS radical cation assay and daidzein > genistein on DPPH assay, respectively. Daidzin and genistin had weak antioxidant activity on DPPH radical scavenging.

Liposome protection was used as an index to assay the protective effects of the WEFM and WEGT on lipid oxidation. As shown in Table 3, WEFM in the range of 50–100 *μ*g/mL exhibited a dose-dependent protective effect, 46.7–67.5%, on the liposome damage induced by the Fe^3+^/H_2_O_2_ reaction. The lipid oxidation inhibition of WEFM at the concentration of 50 *μ*g/mL was about 1.9-fold greater than that of WEGT. Similarly, Table 3 shows that the reference constituents at 50–100 *μ*g/mL possessed protective effects against liposome oxidation. In particular, genistein showed the highest level of protection among the four reference constituents.

 On the other hand, it has been suggested that an antioxidant capacity is due to the development of a reducing ability when reactiing with free radicals and terminating the radical chain reaction [26]. According to Table 3, the reducing power of WEFM followed a concentration-dependent manner, and WEFM showed a higher reducing ability than WEGT. The reducing activities of genistein, genistin, daidzein, and daidzin at 100 *μ*g/mL were 13.7, 13.4, 6.3, and 6.9 *μ*g VitC equivalents/mL. Genistein and genistin had stronger reducing activities than daidzein and daidzin. It was observed that the total phenolics of WEFM had a good correlation with its liposome protection activity and reducing power. This implied that the total polyphenols of WEFM could contribute to the liposome protection and reducing activity. Finally, WEFM did show the protective capacity against lipid molecule oxidation.

In addition, the tyrosinase inhibitory activities of WEFM and WEGT are compared in Table 4. In the range of 50–100 *μ*g/mL, WEFM and WEGT exhibited 26.3–45.8% and 11.7–18.9% inhibitory effects on tyrosinase activity, respectively. The tyrosinase inhibition of WEFM at the concentration of 100 *μ*g/mL was about 2.4-fold greater than that of WEGT. In this test, the tyrosinase inhibitory effects of WEFM increased with an increase in sample concentration and were superior to that of WEGT. Similarly, Table 4 shows that the four marker compounds possess inhibitory effects against tyrosinase. The tyrosinase inhibition of genistein, genistin, daidzein, and daidzin was 58.6, 53.3, 55.8, and 35.8% at 100 *μ*g/mL. Previous reports have suggested that metal chelators could be used as tyrosinase inhibitors [22]; thus, the metal chelating capacity of WEFM and WEGT was determined. As shown in Table 4, the metal chelating capacity of WEFM and WEGT was 38.3% and 16.8% at 100 *μ*g/mL, respectively. This result showed that WEFM exhibited a greater inhibitory effect on chelating activity than that of WEGT. The chelating effects of genistein, genistin, daidzein, and daidzin at 100 *μ*g/mL were 42.6, 48.8, 77.8, and 75.8%, respectively. Genistein and genistin had weaker chelating activity than daidzein and daidzin. However, daidzin, with the tyrosinase inhibition effect of 35.8% at 100 *μ*g/mL, showed the lowest level of inhibition among the four reference constituents.

## 4. Discussion

The destructive stress derived from reactive oxygen species will induce oxidation in biomolecules (e.g., lipids, proteins, and DNA) and aggravate the progression of cells to a pathological status [25]. On the other hand, tyrosinase not only catalyzes unfavourable reactions, which deteriorated since the appearance and nutritional value of foods, but it also increases the oxidative risk in different physiological systems [27]. Recent reports have suggested that phytochemical-rich plants with a complex array of antioxidant exhibit protective effects against harmful stress in various models [24]. From the data of this study, WEFM exhibited a better capacity in radical scavenging, lipid peroxidation prevention, and tyrosinase inhibition than those of WEGT.

Free radicals can induce pathological events such as inflammation, aging, and carcinogenesis [25]. In this study, WEFM showed a significant antioxidant activity. The HPLC chromatogram of WEFM displayed four peaks belonging to phenolic components identified as daidzin, daidzein, genistin, and genistein. The equivalent concentrations of daidzin, daidzein, genistin, and genistein in WEFM were 0.25, 0.33, 8.20, and 8.27 mg/g extract, respectively. These four markers show biological effects including antioxidative activities, anti-LDL oxidation, and anticancer effects [2, 28]. These phenolic components are a popular source of antioxidants, especially genistein, which was one of the highest dietary flavonoids [2, 23]. In this study, genistein showed the highest antioxidant values among four marker compounds by the ABTS, reducing activity and liposome oxidation assay. As shown in Figure 1 and Table 2, genistein and genistin are richer in WEFM and better quenchers against ABTS radical, compared to daidzin and daidzein, which are less potent ABTS scavengers but more efficient against DPPH radical. Therefore, genistein may play a more important role in WEFM's protective effects. Additionally, there are reports that suggest that the antioxidant properties are correlated to the contents of total polyphenols and flavonoids [24, 25]. The data from the present study support the idea that the antiradical activity should not only be attributed to the different classes of phenolic compounds, but probably to the molecular structures of the individual phenolic compounds.

When lipid peroxidation occurs in cell membranes, it releases arachidonic acid, which is responsible for long-term damage to cells. Further, decreasing of low-density lipoprotein oxidation by natural antioxidants would contribute to the prevention of atherosclerosis [1]. As shown in Table 3, in the liposome model, WEFM showed protective activity against the damage caused by lipid oxidation. Obviously, WEFM could exhibit comparable activities in the inhibition of lipid oxidation and thereby protect biomolecules against damage in tissues. In addition, WEFM showed a better reducing ability than WEGT. This may be attributed to reducing compounds present in WEFM. As shown in Table 1, the level of total polyphenols in WEFM (96.47 mg/g extract) was superior to those present in WEGT (70.41 mg/g extract). Consequently, WEFM could react with free radicals to stabilize, terminate harmful chain reactions, and decrease lipid peroxidation. Additionally, four isoflavones, including genistein, genistin, daidzein, and daidzin, also showed potent liposome protection and reducing activity. In general, the antioxidant potency of a given compound is thought to be closely linked to its structural features, such as the orthodihydroxy structure in the B-ring, the 2-3-double bond in conjugation with a 4-oxofunction, and the presence of the 3- and 5-OH functions, or influenced by the presence of glycosidic moieties and position of hydroxyl and methoxy groups. Isoflavone is a potent source of antioxidants, especially genistein, which exerts a number of biological effects such as estrogenic [29], antioxidative, and anticancer effects [30, 31]. Genistein has a 100-fold greater binding affinity than daidzein for the mouse uterine cytosolic estrogen receptors [32]. Thus, WEFM could exhibit a higher reduction activity and liposome protection activity than WEGT due to the higher content of genistein and genistin, total polyphenols, or total flavonoids. 

It has been reported that tyrosinase, also known as a polyphenol oxidase, plays a critical role in catalyzing the melanogenesis and promoting the reactive metabolites produced in the process of melanin formation [27]. Meanwhile, the synthesis of melanin will cause glutathione depletion and the formation of hydrogen peroxide. As shown in Table 4, WEFM clearly decreased tyrosinase activity *in vitro*. This finding suggests that WEFM decreases the level of stress resulting from a reduction in glutathione and the quinone-induced cell damage. Previous studies have supported the role of metal ion chelation in the process of tyrosinase inhibition, and a linear correlation existed between chelating activity and tyrosinase inhibition [22]. Polyphenolic compounds, such as flavonoids, could form complexes with metal ions and exhibit antioxidative action. As shown in Table 1, the level of total flavonoids in WEFM (52.76 mg/g extract) was superior to those present in WEGT (37.91 mg/g extract). In this study, WEFM showed greater inhibitory effects on tyrosinase activity and metal ion chelating than those of WEGT. Moreover, the tested standards, genistein and genistin, are stronger tyrosinase inhibitors than daidzein and daidzin, but lower with their metal ion chelating activities. These findings suggest that WEFM and WEGT exhibited the inhibition of tyrosinase could be associated with their total flavonoids. As shown in Table 1, each of reference compounds is only a very small portion of total. It is possible that the synergistic effects may exist between total flavonoids and four marker compounds of WEFM in tyrosinase inhibitory effects. Other unknown active components present in WEFM and WEGM could also play critical roles in their biological effects.

## 5. Conclusions

Our experimental study revealed that WEFM exerted significant antioxidative effects and antityrosinase activity in the acellular systems of this study. Therefore, the root extract of *Flemingia macrophylla* is interesting as herbal antioxidant for pharmaceutical products and cosmetics.

## Figures and Tables

**Table 1:** The levels of four marker compounds, total polyphenols, and flavonoids in WEFM and WEGT.

Constituents (mg/g extract)	WEFM	WEGT
Daidzin	0.25 ± 0.03	1.34 ± 0.05
Daidzein	0.33 ± 0.04	4.23 ± 0.02
Genistin	8.20 ± 0.11	0.16 ± 0.04
Genistein	8.87 ± 0.06	0.21 ± 0.06
Total polyphenols^a^	96.47 ± 1.58	70.41 ± 0.83
Total flavonoids^b^	52.76 ± 0.17	37.91 ± 0.32

The data were displayed with mean ± S.D. of three experiments individually and analyzed by ANOVA (*P* < 0.05).

^a^Results are expressed in gallic acid equivalents/g extract.

^b^Results are expressed in rutin equivalents/g extract.

**Table 2:** The radical scavenging activities of WEFM, WEGT, and four marker compounds.

Samples	IC_50_ value (*μ*g/mL)	
	ABTS scavenging	DPPH scavenging
WEFM	23.05 ± 0.96	65.28 ± 1.34
WEGM	146.23 ± 2.56	298.66 ± 3.37
Daidzin	19.23 ± 3.78	>500
Daidzein	9.64 ± 0.36	121.28 ± 0.97
Genistin	8.89 ± 1.05	>500
Genistein	5.02 ± 0.84	162.75 ± 1.45

The data were displayed with mean ± S.D. of three experiments individually and analyzed by ANOVA (*P* < 0.05).

**Table 3:** Effects of WEFM, WEGT, and four marker compounds on reducing activity and liposome protection.

Samples	(*μ*g/m)	Reducing activity	Liposome protection
		(*μ*g VitC/mL)	(%)
WEFM	50	10.2 ± 0.2	46.7 ± 2.1
	100	20.1 ± 0.3	67.5 ± 0.4
WEGM	50	4.5 ± 0.1	24.5 ± 1.5
	100	7.7 ± 0.2	43.3 ± 1.0
Daidzin	50	2.9 ± 0.1	40.0 ± 0.8
	100	4.9 ± 0.1	63.6 ± 0.6
Daidzein	50	5.3 ± 0.2	45.2 ± 1.4
	100	6.3. ± 0.2	59.1 ± 2.1
Genistin	50	9.2 ± 0.4	62.4 ± 1.8
	100	13.4 ± 0.6	74.0 ± 1.1
Genistein	50	9.8 ± 0.5	64.2 ± 2.5
	100	13.7 ± 0.3	78.6 ± 2.8

The data were displayed with mean ± S.D. of three experiments individually and analyzed by ANOVA (*P* < 0.05).

**Table 4:** Effects of WEFM, WEGT, and four marker compounds on chelating activity and tyrosinase inhibition.

Samples	(*μ*g/mL)	Chelating activity	Tyrosinase inhibition
		(%)	(%)
WEFM	50	20.1 ± 1.7	26.3 ± 1.7
	100	38.3 ± 0.7	45.8 ± 0.8
WEGM	50	10.9 ± 1.8	11.7 ± 2.0
	100	16.8 ± 1.1	18.9 ± 1.6
Daidzin	50	44.6 ± 2.2	20.6 ± 1.6
	100	75.8 ± 2.7	35.8 ± 0.7
Daidzein	50	52.0 ± 0.8	30.8 ± 1.2
	100	77.8 ± 2.1	55.8 ± 1.4
Genistin	50	30.7 ± 1.1	28.6 ± 2.2
	100	48.8 ± 0.6	53.3 ± 0.9
Genistein	50	27.3 ± 1.4	33.1 ± 1.4
	100	42.6 ± 2.6	58.6 ± 0.8

The data were displayed with mean ± S.D. of three experiments individually and analyzed by ANOVA (*P* < 0.05).
